# Meta-analysis of the association between adiponectin SNP 45, SNP 276, and type 2 diabetes mellitus

**DOI:** 10.1371/journal.pone.0241078

**Published:** 2020-10-22

**Authors:** Yuwei Dong, Gongping Huang, Xin Wang, Zhaoming Chu, Jingzhi Miao, Houwen Zhou

**Affiliations:** 1 College of Food and Biology Engineering, Xuzhou University of Technology, Xuzhou, Jiangsu, China; 2 Xuzhou Technology Limited Company of United Gene, Xuzhou, Jiangsu, China; Shanghai Jiao Tong University, CHINA

## Abstract

**Objective:**

The present study aimed to determine whether the polymorphisms at rs2241766 and rs1501299 on the *ADIPOQ* gene were related to the susceptibility of type 2 diabetes mellitus (T2DM).

**Methods:**

Eight databases, PubMed, GWAS, Embase, Lochrane, Ebsco, CNKI (Chinese National Knowledge Infrastructure), VIP (Viper Database) and ChinaInfo were searched, and a meta-analysis of susceptibility was conducted between SNP45, SNP276 polymorphisms and T2DM. Furthermore, HWE test was conducted to assess the genetic balance of the study, evaluate the quality of Newcastle–Ottawa quality assessment scale (NOS), and establishing allelic, dominant, recessive, heterozygous, and homozygous gene models.

**Results:**

This meta-analysis included 53 articles, encompassing 9285 cases with rs2241766 and 14156 controls and 7747 cases with rs1501299 and 10607 controls. For the rs2241766 locus, a significant correlation was found in the three models by the subgroup analysis. Western Asians: dominant gene model (TT + TG vs. GG, P = 0.01); heterozygous gene model (TG vs. GG, P = 0.02); homozygous gene model (TT vs. GG, P = 0.01). South Asians: dominant gene model (TT + TG vs. GG, P = 0.004); heterozygous gene model (TG vs. GG, P = 0.009); homozygous gene model (TT vs. GG, P = 0.005). However, no statistically significant correlation was established among the five genetic models for rs1501299 locus.

**Conclusion:**

The findings of the present study indicated that the T allele of rs2241766 polymorphism is the susceptibility locus of T2DM in the West Asian population, but has a protective effect in the South Asian population, albeit further studies are needed in other populations. Also, no association was found between the *ADIPOQ* rs1501299 polymorphism and T2DM.

## Introduction

Diabetes mellitus is a clinical syndrome caused by the interaction between genetic and environmental factors. The absolute or relative deficiency of insulin secretion and the decreased insulin sensitivity of target cells results in a series of metabolic disorders related to glucose, protein, fat, water, and electrolytes. According to the data released by the International Diabetes Federation (IDF), 425 million individuals have diabetes worldwide, of which, >350 million are at high risk. It is estimated that about 700 million individuals would be suffering from diabetes by 2045 [1]. Type 2 diabetes mellitus (T2DM) accounts for the vast majority of diabetes mellitus and is a complex polygenic disease. However, the molecular and genetic mechanisms underlying the gene mutation and gene interaction are yet unclear.

Adiponectin (*ADIPOQ*) regulates fatty acid oxidation, glucose uptake, and glycogenesis, which is related to the pathogenesis of diabetes [2]. Therefore, *ADIPOQ* is a candidate gene for the study of metabolic syndrome and T2DM. Rs2241766 and rs1501299 are crucial loci in the *ADIPOQ* gene. rs2241766 is located in exon 2 of the gene, and its polymorphism might affect the shearing or stability of precursor mRNA or alter the protein level. rs1501299 is located in the second intron of the *ADIPOQ* gene, and its polymorphism might affect the function of the neighboring exon. The polymorphism of these two sites accelerates the occurrence of T2DM and affects the body’s insulin sensitivity [52].

Some studies have shown that rs2241766 [23, 27, 30, 39, 43] and rs1501299 [9, 28–30] polymorphisms are related to T2DM, while others have a contrasting viewpoint: rs2241766 [4, 9, 16, 17, 26, 28, 31, 34, 46–48, 50, 51, 53] and rs1501299 [6, 16, 17, 23, 26, 27, 31, 34, 49, 51–53]. These discrepancies in the results might be attributed to the small size of the population in a single study and the background or ethnic differences of the random samples. Therefore, meta-analysis is essential to determine the correlation between T2DM and gene polymorphism. Fan et al. concluded a meta-analysis, wherein rs1241766 polymorphism significantly increased the risk of T2DM in the Asian population [7]. Han et al. found that SNP45 and SNP276 polymorphism were not associated with T2DM [64]. However, the meta-analysis by Li et al. showed that allele rs2241766 is a T2DM susceptibility gene in the Chinese population, while rs1501299 polymorphism was not associated with T2DM [8]. Currently, the focus on the association of the polymorphism of *ADIPOQ* gene rs241766, rs1501299 to T2DM has increased, and hence, it is necessary to supplement the previous meta-analysis. Some studies have shown that polymorphisms of rs2241766 [15, 21, 49, 55, 56] and rs1501299 [4, 18, 49, 56] may be the influencing factors of T2DM, but other studies demonstrated that neither of the polymorphisms, rs2241766 [5, 11, 32] or rs1501299 [5, 11, 15, 55], was associated with T2DM. Therefore, it is necessary to incorporate recent literature and conduct a meta-analysis.

Many genetic variations are related to the geographic and historical populations that the mutation initially produces, and studies must control population stratification. Although GWAS (Genome-wide association study) reported that *ADIPOQ* gene was associated with T2DM, such as rs266729 and rs6810075 [58, 59], it did not specify the correlation between rs2241766 and rs1501299 and T2DM. Moreover, population stratification is a relatively common source of false positives in GWAS studies. Therefore, meta-analysis is imperative to analyze the subgroups of subjects from different regions and determine the correlation between rs2241766 or rs1501299 and T2DM.

## Materials and methods

### Registration of review protocol

The protocol for this meta-analysis was registered on INPLASY (no. INPLASY202040013) and is available on inplasy.com (https://doi.org/10.37766/inplasy2020.4.0013).

### Search strategy and inclusion criteria

A comprehensive literature search of PubMed, Embase, Lochrane, Ebsco, CNKI (Chinese National Knowledge Infrastructure), VIP (Viper Database), and ChinaInfo was conducted up to November 16, 2019. The subject words, combined with free words, were used in the retrieval strategy. The theme words were “Type 2 Diabetes Mellitus”, “adiponectin”, and “Polymorphism”. More relevant literature had been collected.

The included literature fulfilled the following criteria: (1) The case-control studies or cohort designs on the correlation between the polymorphism of *ADIPOQ* gene rs2241766 or rs1501299 and T2DM. (2) The genotype distribution conformed to the HWE (Hardy-Weinberg Equilibrium) balance in the control population. (3) The genotype frequency, the odds ratio (OR) value, or 95% confidence interval (CI) were directly given in the literature. (4) The language of the literature was Chinese or English. (5) For multiple studies of the same author, the latest study or that with sufficient data was selected. On the other hand, case reports, animal studies, reviews, editorial reviews, and literature with incomplete data were eliminated.

### Data extraction and quality appraisal

The two authors (Gongping Huang and Xin Wang), who conducted the literature search, also extracted the data from the studies independently. Any disagreement was adjudicated by consulting a third author (Yuwei Dong). The following information was extracted from the studies: first author, year of publication, study area, diagnosis criteria for T2DM, data of case and control groups, and HWE test results. The studies were evaluated using the Newcastle–Ottawa quality assessment scale (NOS), with a score range of 0–9; those with a score >4 were qualified.

### Statistical analysis

Revman 5.3 was used to calculate the combined OR value and 95% CI to evaluate the association between the polymorphisms of *ADIPOQ* gene rs2241766 or rs1501299 and T2DM. Random effect model was used to calculate the combined OR value and 95% CI. I^2^ value and Q value were used to test the heterogeneity, and P-value calculated by Z test was used to evaluate the meta-analysis results. The sensitivity was analyzed by the influence of a single study on heterogeneity. Stata12.0 was used to conduct Begg’s funnel plot test and Egger’s test for the meta-analysis of this paper, and then, bias was analyzed and evaluated.

## Results

### Characteristics of eligible studies

**Fig 1:**
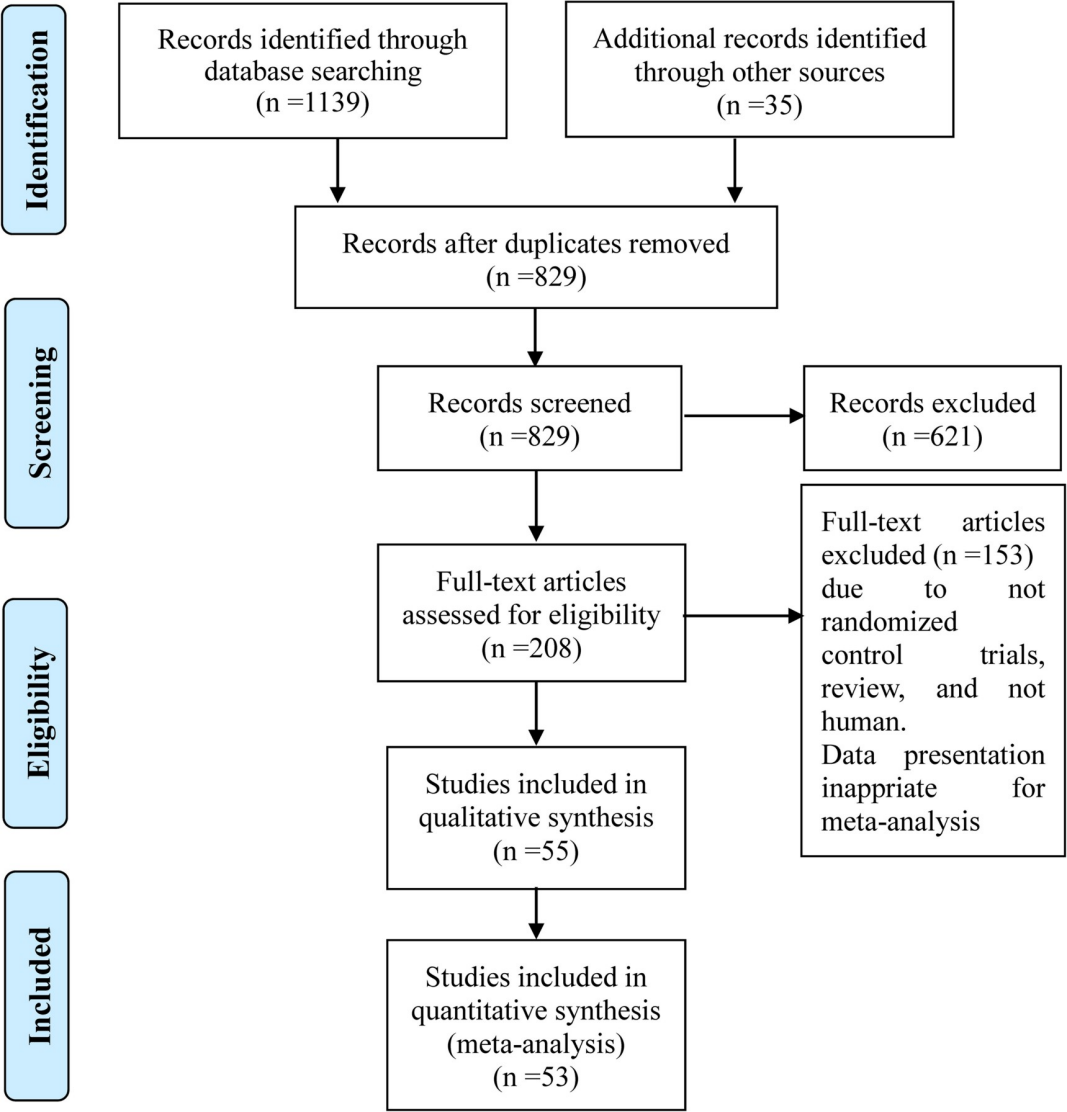
The PRISMA schematic of the meta-analysis.

The initial search retrieved 1,174 potential references. After screening, 53 trials were found to be eligible for the meta-analysis. Fig 1 shows the step-by-step identification and selection process. Table 1 summarizes the main characteristics of the included studies. A total of 53 studies encompassed 9285 cases with rs2241766 and 14156 controls and 7747 cases with rs1501299 and 10607 controls.

**Table 1:** Characteristics of case-control studies included in the meta-analysis.

Authors	Year	Country	Ethnicity	SNPs	diagnosis	Genotyping Method	P-value for HWE	NOS
Aioanei et al. [11]	2019	Eastern European	Europe	rs1501299	NA	PCR-RFLP	0.5318	6
Atsushi et al. [5]	2017	Japan	East Asia	rs2441766	NA	Taqman technology	0.9566	6
				rs1501299			0.8291	
Benedetta et al. [9]	2015	Italian	Europe	rs2441766	NA	Taqman technology	0.7346	7
				rs1501299				
Claudia et al. [13]	2002	America	Europe	rs2441766	WHO	AS-PCR	0.2618	5
Dong et al. [51]	2004	China	East Asia	rs2241766	WHO	PCR-RFLP	0.6746	6
				rs1501299			0.8393	
Frank et al. [19]	2004	America	America	rs1501299	ADA	Real-time PCR	0.5518	8
Fumeron et al. [20]	2004	French	Europe	rs2241766	ADA	PCR-molecular Beacon	0.6811	6
Gable et al. [14]	2007	UK	Europe	rs1501299	WHO	Real-Time PCR	0.9912	7
Hailal et al. [21]	2014	Turkey	West Asia	rs241766	ADA	PCR-RFLP	0.3490	6
Hannan et al. [15]	2016	Bahrain	West Asia	rs1501299	ADA	PCR-RFLP	0.4802	7
Hao et al. [26]	2009	China	East Asia	rs2241766	WHO	PCR-RFLP	0.4678	5
				rs1501299			0.8665	
He et al. [24]	2012	China	East Asia	rs2241766	WHO	PCR-RFLP	0.6234	5
Ina et al. [25]	2012	Romania	Europe	rs1501299	NA	PCR-RFLP	0.1583	8
Ji et al. [56]	2015	China	East Asia	rs2241766	WHO	PCR-RFLP	0.1580	6
				rs1501299			0.0635	
Ji et al. [6]	2018	Korea	East Asia	rs2241766	ADA	Real-time PCR	0.8655	7
				rs1501299			0.7300	
Jose´ L et al. [27]	2005	Spain	Europe	rs2241766	WHO	SNaPshot	0.4431	5
Kang et al. [47]	2012	China	East Asia	rs2241766	ADA	PCR-RFLP	0.3606	8
Kang et al. [57]	2013	China	East Asia	rs1501299	WHO	PCR-DS	0.4813	6
Lee et al. [48]	2005	Korea	East Asia	rs2241766	WHO	SNaPshot	0.2724	6
				rs1501299			0.0575	
Li et al. [53]	2010	China	East Asia	rs1501299	WHO	PCR-RFLP	0.0999	6
Lin et al. [12]	2012	China	East Asia	rs1501299	NA	PCR-RFLP	0.5245	7
Madhukar et al. [35]	2012	India	South Asia	rs2241766	NA	PCR-RFLP	0.1039	7
Magdlena et al. [30]	2009	Poland	Europe	rs2241766	WHO	PCR-RFLP	0.7721	6
Marcio et al. [31]	2010	Japan	East Asia	rs2241766	WHO	PCR-DS	0.5647	7
				rs1501299			0.3297	
Monica et al. [33]	2006	Mexico	America	rs2241766	ADA	PCR-SSCP	0.7800	5
Nasser et al. [34]	2012	Saudi Arabia	South Asia	rs1501299	WHO	PCR-RFLP	0.5212	7
							0.6504	
Olavi et al. [36]	2005	Finland	Europe	rs2241766	NA	PCR-RFLP	0.3968	6
Ozra et al. [37]	2010	Iran	West Asia	rs2241766	ADA	PCR-RFLP	0.4548	6
Populaire et al. [10]	2003	Japan	East Asia	rs2441766	NA	PCR-DS	0.5028	5
				rs1501299			0.0852	
Potapov et al. [45]	2008	Russia	Europe	rs2241766	WHO	PCR-RFLP	0.8475	5
Razwa et al. [40]	2016	Mangladesh	South Asia	rs2241766	WHO	PCR-RFLP	0.6594	5
Ruhi et al. [41]	2014	India	South Asia	rs2241766	NA	PCR-RFLP	0.7028	6
Schwarz et al. [38]	2006	Germany	Europe	rs2241766	WHO	Real-time PCR	0.8809	5
Sheng et al. [44]	2016	China	East Asia	rs2241766	NA	Taqman technology	0.8420	6
				rs1501299			0.6949	
Shi et al. [46]	2007	China	East Asia	rs2241766	WHO	PCR-RFLP	0.6897	7
Shirin et al. [42]	2011	Iran	West Asia	rs2241766	ADA	PCR-RFLP	0.0954	6
Sun et al. [55]	2014	China	East Asia	rs1501299	WHO	PCR-RFLP	0.9824	6
Takeuchi et al. [16]	2008	Japan	East Asia	rs2441766	WHO	Taqman technology	0.6785	5
				rs1501299			0.6128	
Tsai et al. [32]	2014	China	East Asia	rs2241766	NA	AS-PCR	0.5399	5
				rs1501299			0.0942	
Vasseur et al. [17]	2005	French	Europe	rs2441766	WHO	PCR-DS	0.1381	6
				rs1501299			0.3456	
Wan Ching Toy [3]	2011	Singapore	South Asia	rs2241766	WHO	Real-time PCR	0.0707	7
Wang et al. [54]	2005	China	East Asia	rs2241766	WHO	PCR-RFLP	0.1590	6
Wang et al. [43]	2007	China	East Asia	rs1501299	WHO	Real-time PCR	0.1138	6
Wang et al. [52]	2008	China	East Asia	rs1501299	ADA	PCR-SSCP	0.1627	6
Wang et al. [29]	2009	China	East Asia	rs1501299	WHO	PCR-RFLP	0.0675	6
Wang et al. [50]	2009	China	East Asia	rs2241766	WHO	AS-PCR	0.2484	5
				rs1501299			0.6179	
Xia et al. [23]	2004	China	East Asia	rs2241766	WHO	PCR-RFLP	0.1243	6
				rs1501299			0.1243	
Xu et al. [49]	2018	China	East Asia	rs2241766	Standards of care for type 2 diabetes in China (2013)	PCR-RFLP	0.6906	7
Ye et al. [22]	2009	China	East Asia	rs2241766	ADA	PCR-RFLP	0.3448	8
Ye et al. [4]	2014	China	East Asia	rs2441766	WHO	PCR-RFLP	0.7514	7
				rs1501299			0.1444	
Zhang et al. [28]	2007	China	East Asia	rs2241766	WHO	PCR-RFLP	0.4698	7
				rs1501299			0.0653	
Zhao et al. [18]	2016	China	East Asia	rs1501299	WHO	Real-time PCR	0.0694	8
Zhou et al. [39]	2009	China	East Asia	rs2241766	WHO	PCR-RFLP	0.0782	6

Abbreviations: HWE, Hardy–Weinberg equilibrium; WHO, World Health Organization; ADA, American Diabetes Association

### Meta-analysis

S1 Fig demonstrates the forest plot of the association between the *ADIPOQ* rs2241766 polymorphism and T2DM in each study.

For European population, allele model (T *vs*. G): I^2^ = 0%, OR = 1.13, 95% CI = 1.00–1.28, P = 0.06; dominant gene model (TT + TG *vs*. GG): I^2^ = 0%, OR = 1.20, 95% CI = 0.80–1.79, P = 0.38; recessive gene model (TT *vs*. GG + TG): I^2^ = 0%, OR = 1.14, 95% CI = 0.99–1.31, P = 0.07; TG *vs*. GG: I^2^ = 0%, OR = 1.08, 95% CI = 0.71–1.64, P = 0.72; TT *vs*. GG: I^2^ = 0%, OR = 1.23, 95% CI = 0.82–1.85, P = 0.31.

For East Asian population, allele model (T *vs*. G): I^2^ = 0%, OR = 0.99, 95% CI = 0.93–1.05, P = 0.81; dominant gene model (TT + TG *vs*. GG): I^2^ = 0%, OR = 0.99, 95% CI = 0.87–1.13, P = 0.89; recessive gene model (TT *vs*. GG + TG): I^2^ = 0%, OR = 0.99, 95% CI = 0.92–1.08, P = 0.87; heterozygous gene model (TG *vs*. GG): I^2^ = 0%, OR = 0.99, 95% CI = 0.84–1.14, P = 0.91; homozygous gene model (TT *vs*. GG): I^2^ = 5%, OR = 0.99, 95% CI = 0.85–1.15, P = 0.85.

For West Asian population, the allele model (T *vs*. G): I^2^ = 0%, OR = 1.22, 95% CI = 0.97–1.54, P = 0.09; dominant gene model (TT + TG *vs*. GG): I^2^ = 0%, OR = 2.28, 95% CI = 1.21–4.28, P = 0.01; recessive gene model (TT *vs*. GG + TG): I^2^ = 0%, OR = 1.13, 95% CI = 0.86–1.48, P = 0.38; heterozygous gene model (TG *vs*. GG): I^2^ = 0%, OR = 2.22, 95% CI = 1.14–1.32, P = 0.02; homozygous gene model (TT *vs*. GG): I^2^ = 0%, OR = 2.29, 95% CI = 1.21–4.34, P = 0.01.

For South Asian population, the allele model (T *vs*. G): I^2^ = 25%, OR = 0.83, 95% CI = 0.66–1.05, P = 0.12; dominant gene model (TT + TG *vs*. GG): I^2^ = 0%, OR = 0.53, 95% CI = 0.34–0.82, P = 0.004; recessive gene model (TT *vs*. GG + TG): I^2^ = 6%, OR = 0.90, 95% CI = 0.71–1.14, P = 0.38; heterozygous gene model (TG *vs*. GG): I^2^ = 0%, OR = 0.54, 95% CI = 0.34–0.86, P = 0.009; homozygous gene model (TT *vs*. GG): I^2^ = 0%, OR = 0.53, 95% CI = 0.34–0.83, P = 0.005.

S2 Fig shows the forest plot of the association between the *ADIPOQ* rs1501299 polymorphism and T2DM in each study. The association between the *ADIPOQ* rs1501299 polymorphism and susceptibility to T2DM was observed in the allelic model (T *vs*. G: I^2^ = 40%, OR = 0.99, 95% CI = 0.94–1.04, P = 0.58), dominant model (TT+TG *vs*. GG: I^2^ = 0%, OR = 1.00, 95% CI = 0.90–1.11, P = 0.96), recessive model (TT *vs*. GG+ TG: I^2^ = 42%, OR = 0.98, 95% CI = 0.91–1.04, P = 0.45), heterogeneous model (TG *vs*. GG: I^2^ = 0%, OR = 1.01, 95% CI = 0.91–1.13, P = 0.82), homogeneous model (TT *vs*. GG: I^2^ = 14%, OR = 0.99, 95% CI = 0.89–1.11, P = 0.92).

According to the above meta-analysis, the rs2241766 locus of *ADIPOQ* gene did not show any correlation with T2DM in European and East Asian populations, while that in the Western and South Asian populations was linked to T2DM. Moreover, the rs1501299 locus of the *ADIPOQ* gene was correlated with T2DM in all populations.

### Evaluation of publication bias and sensitivity

**Fig 2:**
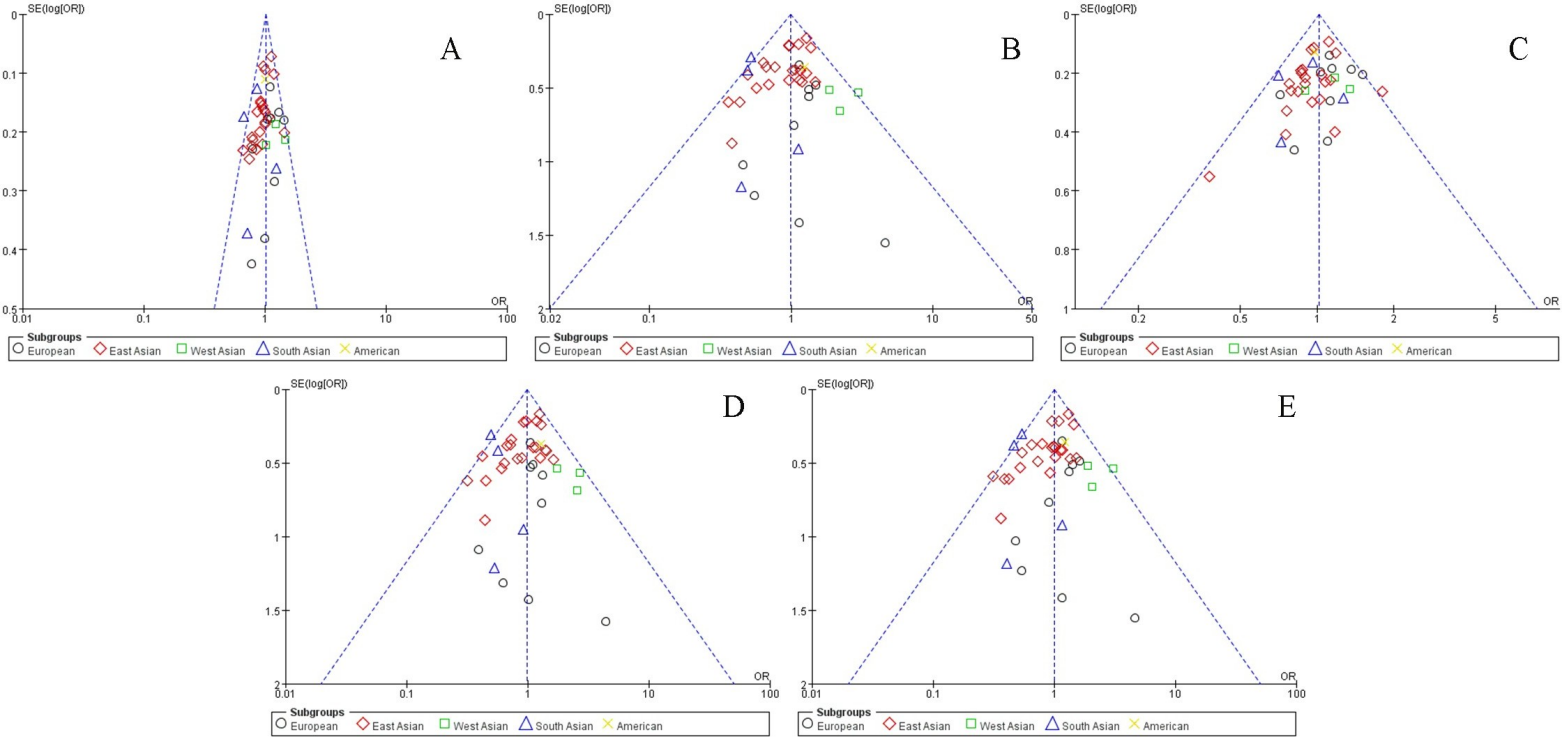
Publication bias indicated by the funnel plots (rs2214766) in an allelic model (A), dominant model (B), recessive model (C), heterogeneous model (D), and homogeneous model (E).

**Fig 3:**
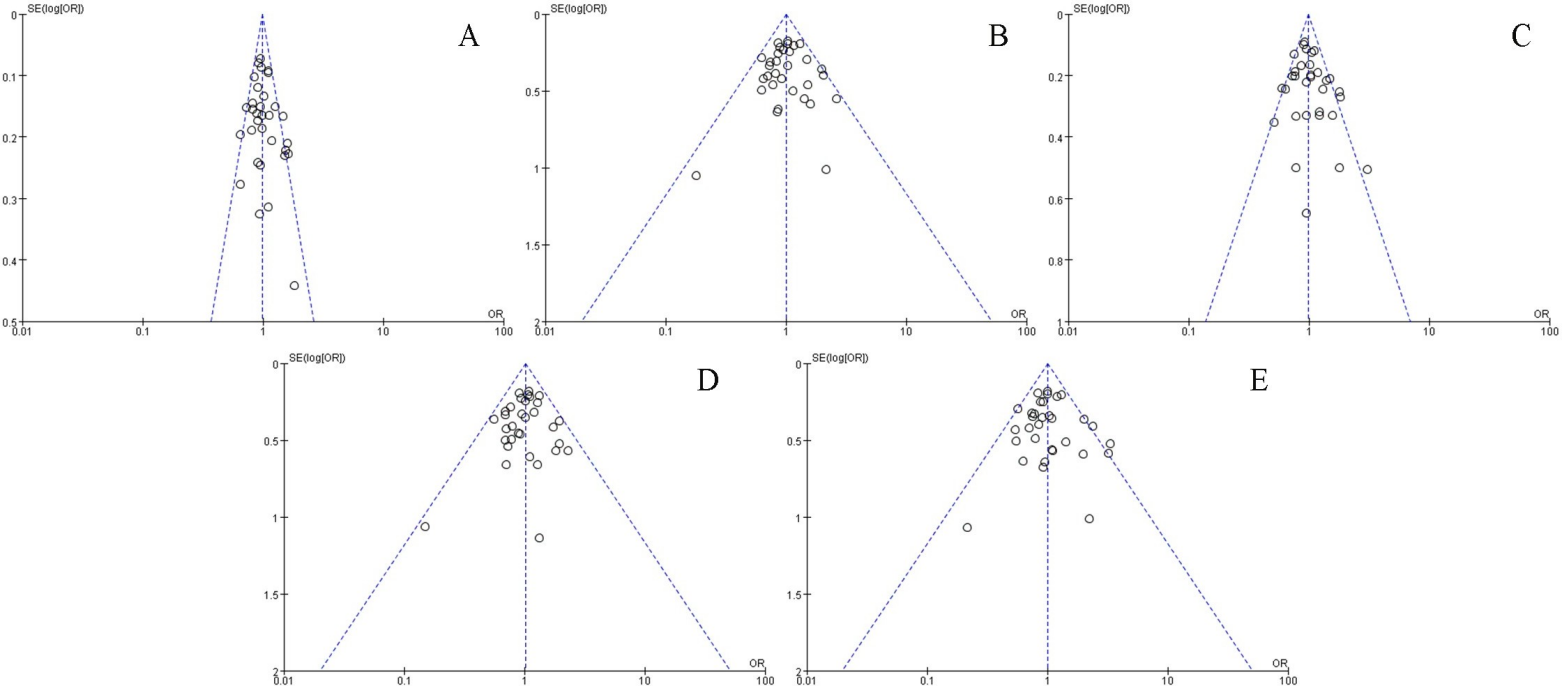
Publication bias indicated by the funnel plots (rs1501299) in an allelic model (A), dominant model (B), recessive model (C), heterogeneous model (D), and homogeneous model (E).

The effect value in each study forms the abscissa, and the reciprocal of standard error is the ordinate while constructing the funnel plot (Figs 2 and 3). The results of Begg’s and Egger’s tests are shown in Table 2. The points of independent study effect value or subgroups included in the meta-analysis were scattered around the center of the funnel plot, indicating the lack of any significant publication bias.

**Table 2:** Begg’s test and Egger’s test results of *ADIPOQ* gene rs2241766, rs1501299 and T2DM.

Model	allelic model	dominant model	recessive model	heterogeneous model	homogeneous model
rs2241766 and T2DM					
Begg’s Test	0.276	0961	0.127	0.809	0.717
Egger’s Test	0.119	0.338	0.126	0.325	0.174
rs1501299 and T2DM					
Begg’s Test	0.189	0.961	0.263	0.758	0.466
Egger’s Test	0.252	0.997	0.175	0.543	0.688

P>0.1

After eliminating the included studies, the combined value and 95% CI evaluation sensitivity were recalculated. The results showed that any single study does not affect the conclusion, thereby proving that the results of the current meta-analysis are stable and reliable.

## Discussion

Diabetes is a metabolic disease characterized by chronic hyperglycemia. More than 90% of the patients are T2DM. Insulin resistance (IR) plays a major role in the pathogenesis of T2DM, and *ADIPOQ* participates in the process of insulin resistance [60]. A specific number of allelic SNPs are present in each region of the *ADIPOQ* gene sequence. Genome-wide analysis showed that 40 gene loci are related to the pathogenesis of T2DM [15]. rs2241766 and rs1501299 are the most frequently studied *ADIPOQ* gene polymorphisms. Hara et al. [61] demonstrated that the G allele frequency of rs2241766 and rs1501299 in Japanese patients with T2DM was higher than that of the T allele and that individuals with rs2241766 and rs1501299 as GG homozygotes had an increased risk of diabetes. However, Menzaghi et al. [62] found that the T allele economic factor of rs2241766 in Caucasians was related to T2DM. According to the study by Min et al. [63], the differences in the association between *ADIPOQ* gene polymorphisms and T2DM are mainly due to the sample size, ethnic diversity, the interaction between gene mutations and environmental factors, and the variations in experimental design and environment. Thus, we analyzed rs2241766 and rs1501299 from the perspective of ethnic differences.

The current meta-analysis showed that the association between the rs2241766 polymorphism of the *ADIPOQ* gene and T2DM might be regional. In Europe and East Asia, no correlation was established between the rs2241766 polymorphism of *ADIPOQ* gene and T2DM, while in West and South Asia, a significant statistical correlation was established in the dominant, heterozygous, and homozygous gene models. The presence of T alleles increases the incidence of T2DM in the West Asian population but protects the South Asian population. However, no correlation was found between the rs1501299 polymorphism of the *ADIPOQ* gene and T2DM, which was consistent with the results of the study by Han et al. [64].

The present study showed regional differences in the association between the rs2241766 polymorphism of the gene and T2DM. Moreover, no correlation was established between the rs2241766 polymorphism of the *ADIPOQ* gene and T2DM in the European population, which was consistent with the results of the study by Potapov et al. [45] in the Russian population. Also, other previous meta-analyses [7, 64] showed that the polymorphism of this site was not associated with T2DM. Secondly, no correlation was established between the rs2241766 polymorphism of the *ADIPOQ* gene and T2DM in the East Asian population, which was consistent with the results of the study by Kaitsai et al. [6, 32, 50, 65]. Conversely, rs2241766 polymorphism is a potential factor of T2DM as demonstrated previously [66–68]. This phenomenon was excluded in the current study because the data did not conform to HWE. Thirdly, the polymorphism of rs2241766 is associated with T2DM in the West and South Asian populations, as shown by Saleh et al. [40] in Bangladesh and Arikoglu et al. [21] in Turkey. Finally, the study by Li et al. [8] showed that the rs2241766 allele might be responsible for susceptibility to T2DM in the Han ethnicity in China, and the studies by Fan et al. [7] and Han et al. [64] also showed similar results.

Notably, the effect of rs2241766 polymorphisms on T2DM is opposite in South and West Asia, i.e., the presence of T alleles increases the incidence of T2DM in the West Asian population, while protecting the South Asian population. However, the factors for the variations in different regions are yet to be elucidated. One possible reason is that SNP +45T>G is a silent polymorphism. Yang et al. [69] showed that SNP 45 T>G, a synonymous mutation (Gly→Gly) in the exon region, does not alter the sequence of amino acids. This suggested that SNP45 polymorphism might affect the levels of adiponectin by influencing the accuracy of pre-mRNA splicing, which in turn, might cause phenotypic variability (T2DM susceptibility) [70]. Another possible reason is that different genetic admixture and environmental factors among South and West Asian populations included in the current study modulate the effects of SNP 45 polymorphisms on adiponectin levels [71, 72], and then affect the T2DM susceptibility. For example, an increased level of circulating adiponectin was detected in post-menopausal females [73], TT or TG genotype with high BMI was detected in different populations [74, 75], and exercise may modify the adiponectin concentration independently of the gene variants [76]. Therefore, additional studies with a large sample size are essential for evaluating the differences of gene-environmental interactions between South and West Asian populations. On the other hand, Jiang et al. found that increased levels of ferritin affect the T2DM risk of disease, and that T2DM occurrence in female populations in Asia and Europe is highly correlated with ferritin levels [77]; the ferritin levels in the South and West Asian populations may be the opposite cause of the findings in both regions. Thus, a study on ferritin levels or gender in both regions might explain this phenomenon.

In addition, the meta-analysis did not show any association between the rs1501299 polymorphism of *ADIPOQ* gene and T2DM, which was consistent with the results of the study by Wang et al. [11, 12, 50]. Conversely, SNP (rs1501299) has a significant correlation with T2DM in the studies by Wang et al. [78] and Jun et al. [79]. The data did not conform to the HWE test, and hence, these studies were excluded. In the Kyrgyz population [80], a correlation was established between the allele and heterozygous genotype of rs1501299 and T2DM. However, limited statistical data and inconformity with the HWE test led to the exclusion of this study.

Nevertheless, this meta-analysis has some limitations. Although most studies did not consider these confounding factors, the unmeasured factors may affect the association between the observed *ADIPOQ* gene polymorphism and T2DM. On the other hand, the present meta-analysis is not sufficient to elucidate the association between *ADIPOQ* gene polymorphism and T2DM in the American and South and West Asian populations and needs to be expanded to the other two regions for substantiation.

## Conclusion

In summary, this meta-analysis showed that the primary outcomes are the regional associations between the rs2241766 polymorphism of *ADIPOQ* gene and T2DM. The T allele of rs2241766 polymorphism may be the susceptibility locus of T2DM in the West Asian population, but has a protective effect in the South Asian population, while no correlation was established in European and East Asian populations. The secondary outcome is that the rs1501299 polymorphism is not related to T2DM.

## Supporting information

S1 Fig Forest plots of the meta-analysis of the association between ADIPOQ rs2241766 polymorphisms and T2DM in an allelic model (A), dominant model (B), recessive model (C), heterogeneous model (D), and homogeneous model (E).
(TIF)

S2 Fig Forest plots of the meta-analysis of the association between ADIPOQ rs1501299 polymorphisms and T2DM in an allelic model (A), dominant model (B), recessive model (C), heterogeneous model (D), and homogeneous model (E).
(TIF)

S1 File PRISMA checklist. (DOC)

## Abbreviations

T2DM: type 2 diabetes mellitus
CI: confidence interval
NOS: Newcastle–Ottawa Quality Assessment Scale
OR: odds ratio
CNKI: Chinese National Knowledge Infrastructure
VIP: Viper Database
HWE: Hardy-Weinberg Equilibrium
GWAS: Genome-wide association study
