# Supplementary figures and images for: Meta-analysis of the association between adiponectin SNP 45, SNP 276, and type 2 diabetes mellitus
Source: PLoS One. 2020 Oct 22;15(10):e0241078. doi: 10.1371/journal.pone.0241078 (PMC7580922; doi:10.1371/journal.pone.0241078)

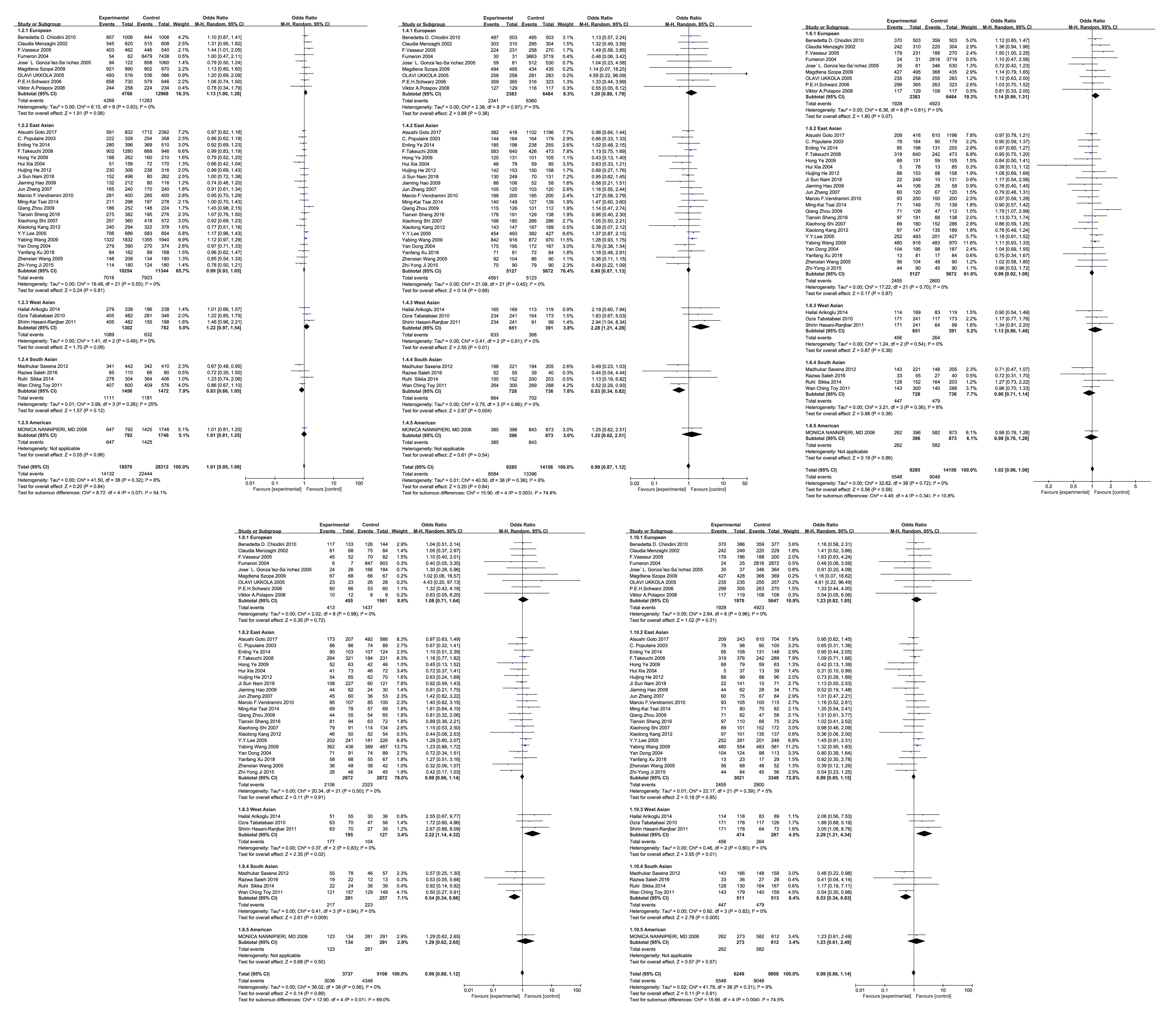

Supplement: S1 Fig — Forest plots of the meta-analysis of the association between ADIPOQ rs2241766 polymorphisms and T2DM in an allelic model (A), dominant model (B), recessive model (C), heterogeneous model (D), and homogeneous model (E). (TIF) [file pone.0241078.s001.tif]

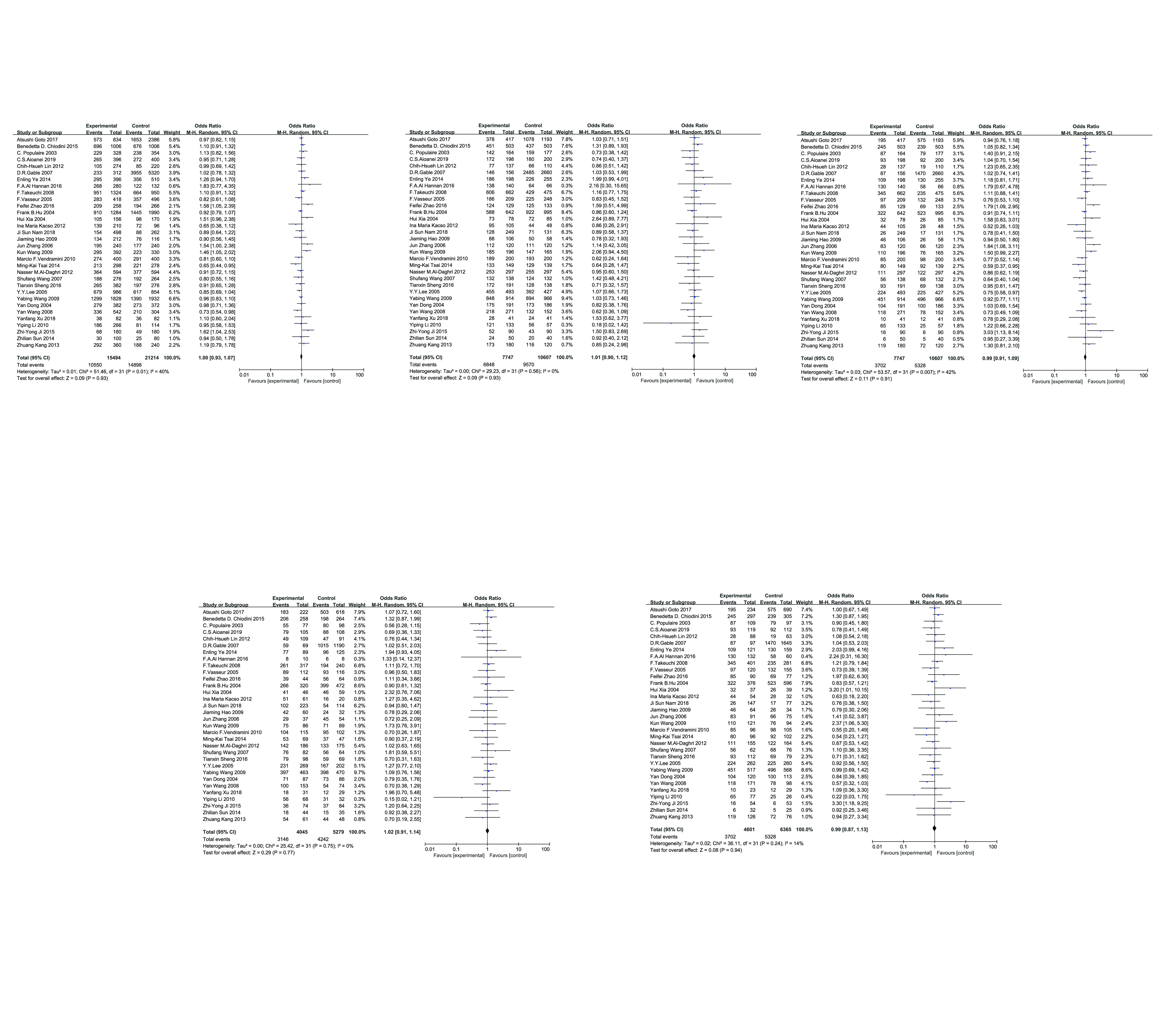

Supplement: S2 Fig — Forest plots of the meta-analysis of the association between ADIPOQ rs1501299 polymorphisms and T2DM in an allelic model (A), dominant model (B), recessive model (C), heterogeneous model (D), and homogeneous model (E). (TIF) [file pone.0241078.s002.tif]
